# Codon optimization of antigen coding sequences improves the immune potential of DNA vaccines against avian influenza virus H5N1 in mice and chickens

**DOI:** 10.1186/s12985-016-0599-y

**Published:** 2016-08-26

**Authors:** Anna Stachyra, Patrycja Redkiewicz, Piotr Kosson, Anna Protasiuk, Anna Góra-Sochacka, Grzegorz Kudla, Agnieszka Sirko

**Affiliations:** 1 Institute of Biochemistry and Biophysics, Polish Academy of Sciences, ul., Pawinskiego 5A, 02-106 Warsaw, Poland; 2 Mossakowski Medical Research Centre Polish Academy of Sciences, ul., Pawinskiego 5, 02-106 Warsaw, Poland; 3 MRC Human Genetics Unit, Institute of Genetics and Molecular Medicine, University of Edinburgh, Edinburgh, EH4 2XU Scotland UK

**Keywords:** Chickens, DNA vaccine, GC content, H5N1, Influenza, Mice

## Abstract

**Background:**

Highly pathogenic avian influenza viruses are a serious threat to domestic poultry and can be a source of new human pandemic and annual influenza strains. Vaccination is the main strategy of protection against influenza, thus new generation vaccines, including DNA vaccines, are needed. One promising approach for enhancing the immunogenicity of a DNA vaccine is to maximize its expression in the immunized host.

**Methods:**

The immunogenicity of three variants of a DNA vaccine encoding hemagglutinin (HA) from the avian influenza virus A/swan/Poland/305-135V08/2006 (H5N1) was compared in two animal models, mice (BALB/c) and chickens (broilers and layers). One variant encoded the wild type HA while the other two encoded HA without proteolytic site between HA1 and HA2 subunits and differed in usage of synonymous codons. One of them was enriched for codons preferentially used in chicken genes, while in the other modified variant the third position of codons was occupied in almost 100 % by G or C nucleotides.

**Results:**

The variant of the DNA vaccine containing almost 100 % of the GC content in the third position of codons stimulated strongest immune response in two animal models, mice and chickens. These results indicate that such modification can improve not only gene expression but also immunogenicity of DNA vaccine.

**Conclusion:**

Enhancement of the GC content in the third position of the codon might be a good strategy for development of a variant of a DNA vaccine against influenza that could be highly effective in distant hosts, such as birds and mammals, including humans.

**Electronic supplementary material:**

The online version of this article (doi:10.1186/s12985-016-0599-y) contains supplementary material, which is available to authorized users.

## Background

Vaccines against influenza are traditionally produced from viruses propagated in chicken embryos, however the first formulations with antigens produced in cell lines are also available, for example Flucelvax®. The traditional techniques have a number of disadvantages: (i) the process is slow and inflexible, which hinders a fast reaction in the case of new outbreaks, (ii) capacity is too small to produce enough doses, (iii) workers are exposed to the dangerous live pathogen and (iv) the virus can mutate during propagation. Moreover, highly pathogenic strains are difficult to propagate in eggs in sufficient amounts, due to their harmful effect on the host, and have to be reassorted or genetically engineered. Problematic are also traces of chicken proteins present in formulation, which are common allergens [1, 2]. The DNA vaccines seem to be a very promising alternative with multiple advantages. They are relatively easy and economical in production due to the lack of long-lasting and complicated procedures of antigen multiplication and purification. They can be quickly re-designed and re-constructed in case of sudden new disease outbreaks. They guarantee antigens with native structure, identical with those within infection, containing all posttranslational modifications, since they are produced *in vivo* in host cells. Moreover, they are safe and no infective form of the pathogen is needed at any step. DNA itself is also more stable in storage and transport than proteins. DNA vaccines induce both humoral and cellular immunological responses, stimulating T cells, antigen presenting cells and antibodies production, ensure broad, long lasting and protective response [3, 4]. Thus, it is not surprising that several clinical trials of DNA vaccines against influenza are now ongoing (http://clinicaltrials.gov/) [5, 6].

The expression level of cDNA encoding an antigen in the cells of immunized host is an important factor influencing the immunological potential of DNA-based vaccines. Manipulations within the coding sequence, such as replacing the rare codons with the synonymous codons preferred by the host organism and avoidance of RNA secondary structures motifs or others unprofitable features have been applied to improve the effectiveness of DNA vaccines against influenza [7]. For example, codon optimization of DNA vaccine based on HAs from A/New Caledonia/20/99 (H1N1) and A/Panama/2007/99 (H3N2) not only enhanced its immunogenicity but also might lead to the reduction of the number of required doses [8]. Similar results were also reported for DNA vaccine based on HA derived from the swine influenza virus A/Texas/05/2009 (H1N1) [9]. These authors demonstrated that optimization of the codon bias of HA from H1N1 resulted in stimulation of CD8+ (determined by the high levels of TNF, IFNγ and IL-2) and in elevated level of antibody production. Recently, also immunization of ponies (mixed breeds of Shetland blood, Welsh blood, Florida swamp pony blood) with monovalent or trivalent DNA vaccines (with mammalian preferred codons) encoding HAs from different strains of H3N8 equine influenza was reported [10]. The vaccine was administered to ponies that were subsequently challenged with the homologues virus. The degree of protection, virus shedding and clinical symptoms after infection were significantly reduced in all immunized groups compared to the negative control. Moreover, a moderate level of cross response was obtained in the group that received the trivalent formulation.

Avian influenza is a serious and highly infectious disease of poultry and other bird species, caused by influenza viruses which can be also transmitted to humans causing high mortality [11, 12]. Therefore, development of effective vaccines against avian influenza is very important. In birds, higher effectiveness of the DNA vaccine based on the HA variant with codons optimized for chicken usage, where the optimized gene shared about 75 % nucleotides with the wild type gene, has been reported by several independent research groups. The examples include chicken [13] and Japanese quails [14] immunization by different variants of H5 HA. The authors mentioned several possible reasons of the observed superiority of the modified plasmid, such as increased expression due to usage of the chicken optimized codons, increased mRNA stability due to increased GC content and increased level of CpG motifs that could act as an adjuvant of immunological responses [13]. In contrast, no significant seroconversion differences between the groups immunized with the optimized and non-optimized variants were observed in the case of the DNA vaccine based on HA from the low pathogenic H6N2 virus [15]. The authors observed high inter-individual variation, possibly due to poor efficiency of the delivery method and/or the huge biological variation of individual responses. The H5 HA variants optimized for human preferred codons were also tested. For example, a large set of different *HA* optimized for human preferred codons was tested in mice and chickens and proven to elicit robust protective immune responses against a broad range of H5 influenza strains [16]. Animals received several multivalent combinations of DNA vaccines. Responses were tested by HI and virus microneutralization tests with homologous and heterologous antigens, as well as by the challenge experiment. The obtained results indicated protection against heterologous strains of highly pathogenic avian influenza H5N1 after vaccination with two doses of DNA vaccine. Another interesting approach was applied by the researchers who used the consensus sequence of H5 HA based on the sequences from 467 different H5 strains, optimized it for mammalian expression (using human preferred codons) and observed not only its high expression level but also strong protective immune response in vaccinated laboratory animals [17].

Most above-mentioned studies confirmed that codon optimization to the codon bias of the host improves the efficacy of DNA vaccine. However, several studies in mammalian cells suggest that increasing the GC content provides better mRNA stability, processing and nucleocytoplasmic transport [18–21]. Because the distribution of GC content among genes is similar in mammals and birds (Additional file 1: Figure S1), we hypothesized that GC content optimization might also lead to improved expression and immunogenicity in birds. The DNA vaccine prepared according to such criterion could be effective in many types of hosts, its design would be simplified and the obtained effects more universal. Therefore, the goal of this study was to evaluate the immunogenicity of the variant of DNA vaccine containing nearly 100 % of codons with GC at the third position in two model animals, mice and chickens, and comparing it to the other vaccine variants. All tested vaccine variants were based on HA from the highly pathogenic avian influenza virus A/swan/Poland/305-135V08/2006 (H5N1).

## Methods

### Plasmids used for DNA vaccination

The HAw/pCI, K3/pCI and GK/pCI plasmids were used for DNA vaccination. The HAw/pCI plasmid contains the nucleotide sequence identical to the region encoding the full-length HA from A/swan/Poland/305-135V08/2006 (H5N1). The K3/pCI and GK/pCI plasmids contain two different nucleotide sequences encoding the same H5 HA protein as HAw/pCI (with the leader peptide) but without the proteolytic cleavage site (341-RRRKKRR-347) between HA1 and HA2 subunits. The sequence encoding HA, present in K3/pCI, was optimized for domestic chicken (*Gallus gallus*) and the codon adaptation index (CAI) reached 0.91. In contrast, the sequence encoding HA, present in GK/pCI was not optimized to any codon bias but it was was modified by changing the nucleotides present in the third positions of the codons to either guanine (G) or cytosine (C). The cDNA of K3 and GK were synthesized by GeneScript (USA; http://www.genscript.com/). Comparison of the *HA* sequences is shown in Additional file 2: Figure S2. The inserts were cloned into MluI and SalI restriction sites of the pCI expression vector (Promega, Wisconsin, USA) downstream of the cytomegalovirus (CMV) promoter and upstream of the SV40 late polyadenylation signal. Plasmids were propagated in DH5α strain of *Escherichia coli* and isolated using NucleoBond® PC 10000 EF Giga-scale purification kit (Macherey-Nagel, Düren, Germany).

### Transfection of mammalian cells

The mouse myoblast cells (2x10^5^ cells; C2C12 line) were transfected with 2 μg of HAw/pCI, K3/pCI, GK/pCI or pCI using Lipofectamine® 3000 Reagent (ThermoFisher Scientific, Waltham, USA) as described by the manufacturer. After 48h the cells were scraped off into the RIPA buffer (ThermoFisher Scientific, Waltham, USA), transferred to the 1.5 ml tubes and frozen in liquid nitrogen, following thawing at 37 °C (three times). The homogenates were centrifuged at 10 000× g for 8 min at 4 °C and the equal amounts of protein extract were analyzed by SDS-PAGE (Nu-Page™ 4-12 % Bis-Tris gel, Invitrogen™, Basel, Switzerland) and Western blotting. The nitrocellulose membranes were blocked in 5 % milk in 1× TBS buffer (50 mM Tris, pH 8, 150 mM NaCl, 1 % Tween 80) and incubated for 1.5h with primary antibody anti – HA (H5) (1:500, ImmuneTechnology, USA), or anti- GAPDH (1:20000, Sigma, St. Louis, USA) and for 1h with secondary antibody (anti-rabbit or anti-mouse IgG (whole molecule) − alkaline phosphatase antibody; Sigma, St. Louise, USA). The enzymatic color reaction was generated using NBT/BCIP Stock Solution (Roche, Switzerland). The bands intensity was compared using the Image J (https://imagej.nih.gov/ij/).

### Immunization of animals

**Fig. 1:**
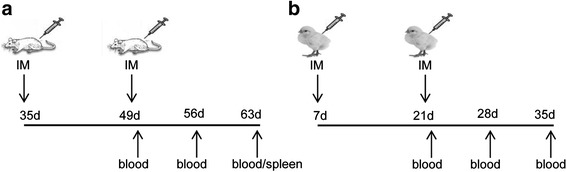
Schedule of mice (**a**) and chickens (**b**) experiments

Vaccine doses were prepared by mixing plasmid DNA (suspended in PBS, pH 7.4) with Lipofectin® (Invitrogen™, Basel, Switzerland) in ratio 6:1 as described earlier [22]. Our previous results indicated that the DNA vaccination was more effective when plasmid was used with a lipid carrier (Additional file 3: Figure S3 and [22]). Schemes of the immunization experiments are presented in Fig. 1 and the number of animals and DNA doses used in each experiment is indicated in Table 1. Specific-pathogen free BALB/c female mice (5–6 weeks of age) were maintained in standard conditions with free access to water and standard mouse diet at the experimental facility in Mossakowski Medical Research Centre Polish Academy of Sciences. Mice were immunized intramuscularly (in quadriceps of left thigh; one spot) and obtained two 50-μl doses of the vaccine, on days 35 and 49 (days of life). Several doses of plasmid DNA were tested in order to choose the most convenient dose for the comparison of the vaccine variants. The blood samples were collected three-fold: two weeks after the first immunization (day 49), one week (day 56) and two weeks (day 63) after the second immunization. Broiler chickens (Ross 308) and layer chickens (Rosa 1) were purchased from commercial brooder on the hatching day and maintained in standard bedding conditions at experimental poultry house. Birds were immunized intramuscularly (in breast muscle, one spot) on days 7 and 21 (day of life) using 60 μg of plasmid DNA mixed with Lipofectin® in a final volume of 100 μl. The dose was chosen as optimal for the purposes of this study based on our previous experiment with chickens immunization [22, 23]. The blood samples were collected from the wing vein on days 21, 28 and 35 in Experiment 1 and on days 21 and 35 in Experiment 2.

**Table 1:** Number of animals used in immunization experiments

		DNA dose	Number of animals per group			
			HAw/pCI	K3/pCI	GK/pCI	pCI
Mice	Experiment 1	20 μg	7	6	-	6
	Experiment 2	10 μg	-	6	7	-
		50 μg	-	6	6	2
	Experiment 3	10 μg	-	6	5	2
	Experiment 4	20 μg	-	6	6	2
Chickens	Experiment 1 (Broilers)	60 μg	8	8	-	5
	Experiment 2 (Layers)	60 μg	-	6	6	2
	Experiment 3 (Broilers)	60 μg	-	10	9	4

All applicable international, national, and/or institutional guidelines for the care and use of animals were followed. The experiments of mice immunization were approved by the Fourth Local Ethical Committee for Animal Experiments at the National Medicines Institutes, Permit Number 03/2014. The experiments with chickens were approved by the Second Local Ethical Committee for Animal Experiments at the Medical University of Warsaw, Permit Number 17/2009.

### Elisa

Mice: 96-well flat-bottom plates (MaxiSorp Surface, Nunc, UK) were coated with 300 ng of purified recombinant H5 HA (A/swan/Poland/305-135V08/2006, H5N1) (derived from a baculovirus system (Oxford Expression Technologies, UK) at 2–8 °C overnight. After removing the coating buffer, the plates were washed three times with 1× PBST (phosphate buffered saline with 0,05 % of Tween-20) and blocked with 2 % of BSA-PBST at 37 °C for 90 min. After 2 washes, the 100-fold in Experiment 1, Experiment 3 and Experiment 4 and the 50-fold (49d) or 200-fold (56d and 63d) in Experiment 2 diluted sera samples were added and incubated at 2–8 °C overnight. Next day, after 4 washes, plates was incubated for 1h at 37 °C with alkaline phosphatase-conjugated goat anti-mouse IgG (Sigma Aldrich, St. Louise, USA). The enzymatic color reaction was performed using alkaline phosphatase yellow (pNPP) liquid substrate (Sigma, St.LouiseUSA), stopped with 3M NaOH and measured (OD_405_) using a Synergy/HT microplate reader (BioTek Instruments, Inc.).

Chickens: 96-well flat-bottom plates (MediSorp Surface, Nunc, UK) were coated with the same antigen as above. After removing the coating buffer, the plates were washed four times with 1× PBST and blocked with 2 % of BSA-PBST at 37 °C for 90 min. Following 2 washes, the 200-fold diluted sera samples were added and incubated at 2–8 °C overnight. Next day, after 5 washes plates were incubated for 1h at 37 °C with peroxidase-conjugated goat anti-chicken IgY (Life Technologies, USA). The enzymatic color reaction was generated using TMB substrate (Sigma, St.Louise, USA), stopped with 0.5M H_2_SO_4_ and measured (OD_450_) as above.

### Antibody endpoint titers

For determination of IgY endpoint titers two-fold serial dilutions of chicken sera collected on day 35 (in range from 10^−3^ to 10^−6^) were made and analyzed using the ELISA protocol. Based on OD_450_ values the absorption curves were made and the endpoint titers were determined using Gen5 Data Analysis Software (BioTek Instruments, Inc.).

### Hemagglutination inhibition (HI)

HI tests were performed according to the OIE standard procedures using the commercially available hemagglutinating antigen prepared from low pathogenic H5N2 strain A/chicken/Belgium/150/1999 (DG Deventer, Netherlands) with 96 % protein sequence similarity to the vaccine antigen. For the HI test, serum to be tested was serially two-fold diluted (1:8 to 1:512) in 25 μl of PBS in V-bottom microtiter plates and an equal volume of HA antigen containing 4 HA units was added. After incubation at room temperature (RT) for 25 min, 25 μl of a 1 % suspension of hens’ red blood cells was added and incubated for 25 min at RT. HI titers are shown as the reciprocal of the highest dilution of sera that completely inhibited hemagglutination.

### Cytokine production assay

Immunized and control mice were euthanized two weeks after boost dose (day 63) and their spleens were harvested. The spleen cell suspensions from the pooled two spleens (from two randomly selected mice from the same group) were washed in RPMI-1640 medium (Sigma, USA) and treated for 5 min with the Lysis buffer (BD, Franklin Lakes, USA) in order to clear red blood cells. To determine the amount of cytokines in culture supernatants, splenocytes (2x10^6^ per well) were incubated in 96-well plates (Corning, Corning, USA) with complete RPMI-1640 with 10 μg/ml of recombinant H5 HA protein purified from the baculovirus system (Oxford Expression Technologies, England), 5 μg/ml Concavaline A (Con A) or medium alone (see above). Cells were incubated for 72h (37 °C, 5 % CO_2_) and centrifuged (10 min, 1000 rpm, 4 °C). The level of cytokines was quantified in the collected supernatants using Cytometric Bead Array Mouse Th1/Th2/Th17 Cytokine Kit (BD, Franklin Lakes, USA) according to the manufacturer’s instructions and the FASCCalibur™ flow cytometer (BD, Franklin Lakes, USA).

### Statistical analysis

Non-parametric tests, such as Kruskal-Wallis (for comparison of multiple groups) or Wald-Wolfowitz, Kolmogorov-Smirnov and Mann-Whitney U (for comparison of two groups) that are components of Statistica 12 (StatSoft, Poland) were used to evaluate the statistical differences. The groups were considered significantly different if at last one of the test was positive (*p* < 0.05).

## Results

### Verification of the HA expression cassettes in mammalian cells

**Fig. 2:**
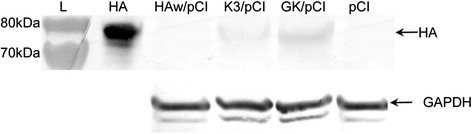
Expression of H5 HA in the C2C12 cell line (mouse myoblasts) transfected with the indicated plasmids: HAw/pCI, K3/pCI, GK/pCI and empty pCI as a negative control. About 200 ng of recombinant H5 HA [A/Bar-Headed Goose/Qinghai/12/05 (H5N1)] was used as a positive control (HA). GAPDH (Glyceraldehyde-3-phosphate dehydrogenase) represents the product of an intrinsic gene, was used as a loading control. Data are representative of at least three independent experiments. L – Page Ruler™Prestaned Protein Ladder with the size of protein bands indicated

The HAw/pCI, K3/pCI and GK/pCI plasmids, containing the variants of H5 HA from A/swan/Poland/305-135V08/2006 (H5N1), were transiently transfected into the mammalian cells and the level of H5 HA protein produced by the transfected cells was monitored (Fig. 2). The cells transfected with GK/pCI usually produced about 15–30 % more HA than the cells transfected with K3/pCI, while the HA protein in cells transfected with HAw/pCI was hardly detectable. These results indicated that enriching nucleotide sequence encoding HA with GC in the third positions of the codons improves the functionality of the HA cassette in mice cells in comparison to the variant optimized for chicken codon bias.

### Comparison of the effectiveness of HAw/pCI and K3/pCI in two animal models

**Fig. 3:**
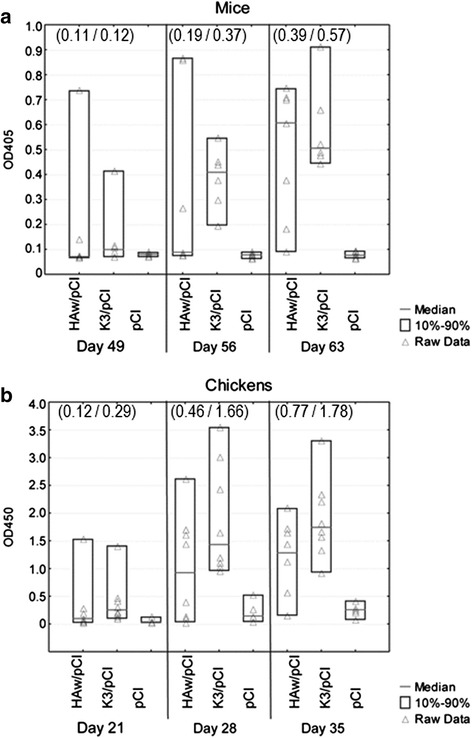
Comparison of humoral responses in animals immunized with two variants of the DNA vaccine. The mice (**a**) and chickens (**b**) sera was tested for the presence of the anti-H5 HA antibodies after immunization with the variant containing the wild type H5 *HA* (HAw/pCI) and the variant containing H5 *HA* optimized to the chicken codon bias (K3/pCI). The results of one-dilution ELISA for individuals, medians and the 10^th^ and 90^th^ percentiles are shown for each group. The number of individuals was as indicated in Table 1, in Experiment 1 for mice and chickens, respectively. The values in the brackets are the geometric means calculated for the K3/pCI and GK/pCI groups

The effectiveness of two DNA vaccines, the wild type (HAw/pCI) and the variant optimized for chicken codon bias (K3/pCI), was compared in two model animals, mice and chickens (broilers). The DNA doses and the number of animals vaccinated with the compared variants is shown as Experiment 1 for either mice or chickens in Table 1. The level of anti-H5 HA antibodies in sera collected from the immunized mice and chickens are shown in Fig. 3a and b, respectively. Both vaccine plasmids stimulated anti-H5 HA humoral response with apparently better parameters in the case of K3/pCI groups, however the differences between K3/pCI and HAw/pCI failed to pass the statistical significance test. In both immunized organisms the geometric means and/or medians of the K3/pCI groups were usually higher than of the respective HAw/pCI groups, especially in sera collected one week after the booster (day 56) for mice and one and two weeks after the booster (days 28 and 35) for chickens. Results of this experiment failed to indicate significant differences between HAw/pCI and K3/pCI, however they might suggest that HAw/pCI is slightly inferior in comparison to K3/pCI in both animal models.

### Mice response to the optimized variants of DNA vaccine

**Fig. 4:**
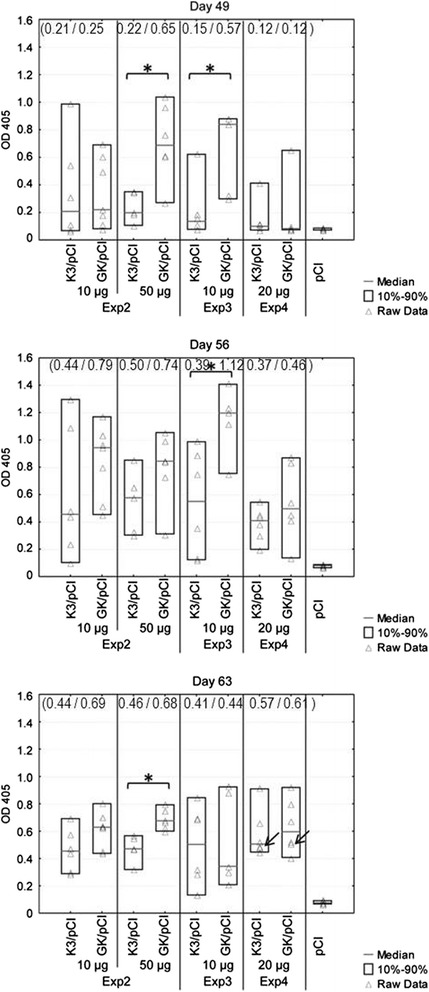
Comparison of humoral responses in mice immunized with two optimized variants of DNA vaccine. The presence of anti H5 HA antibodies was monitored in sera collected from the animals on the indicated days in three independent experiments, Experiments 2, 3 and 4 (see also Table 1). The results for individuals, medians and the 10^th^ and 90^th^ percentiles are shown for each group. Statistically significant differences between groups K3/pCI and GK/pCI (*p* < 0.05) are marked by asterisks. The arrows show from which individuals the spleens have been harvested. The values in the brackets are the geometric means calculated for the K3/pCI and GK/pCI groups

The subsequent mice immunizations have been conducted according to the same general scheme (Fig. 1a) in three independent experiments, labeled as Experiment 2, 3 and 4. The results of one-dilution ELISA, detecting presence of specific anti-H5 HA antibodies in mice sera are shown in Fig. 4. The used doses of DNA and the number of animals in each group are indicated in Table 1. The lowest dose of the DNA (10 μg) was used two times, once in Experiment 2 and once in Experiment 3 but the statistically significant differences between K3/pCI and GK/pCI groups were observed only in Experiment 3, on days 49 and 56. Each of the higher doses of DNA, 50 μg and 20 μg, were tested only once, in Experiment 2 and 4, respectively. The statistically significant differences between the K3/pCI and GK/pCI groups were observed only in case of 50μg dose on days 49 and 63 (Fig. 4).

**Fig. 5:**
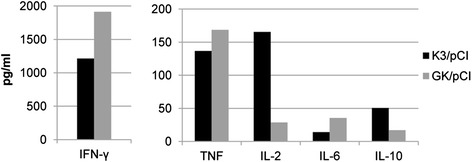
Levels of selected cytokines produced by H5 HA-stimulated splenocytes. The assay was performed with the splenocytes isolated from two pulled down spleens (from two individuals from the same group, indicated by arrows in Fig. 4) collected at the end of experiment (day 63)

The supernatants of liquid cultures of stimulated splenocytes from the spleens isolated at the end of the Experiment 4 (day 63) from two individuals per group were used to measure the levels of interleukin 2 (IL-2), interleukin 4 (IL-4), interleukin 6 (IL-6), interferon-γ (IFN-γ), tumor necrosis factor (TNF), interleukin 17A (IL-17A), and interleukin 10 (IL-10) in a single sample (Fig. 5). The IL-4 and IL-17A were not detected in the tested supernatants. No secretion of TNF, IL-6 and IL-2 or very low secretion IFN-γ was observed in the case of activated splenocytes collected from the control group vaccinated with the empty pCI vector (data not shown). Interestingly, the level of IL-2 and IL-10 was several-fold higher in the case of K3/pCI than GK/pCI, while the levels of IFN-γ, TNF and IL-6 were higher in the case of GK/pCI.

### Chickens response to the optimized variants of DNA vaccine

**Fig. 6:**
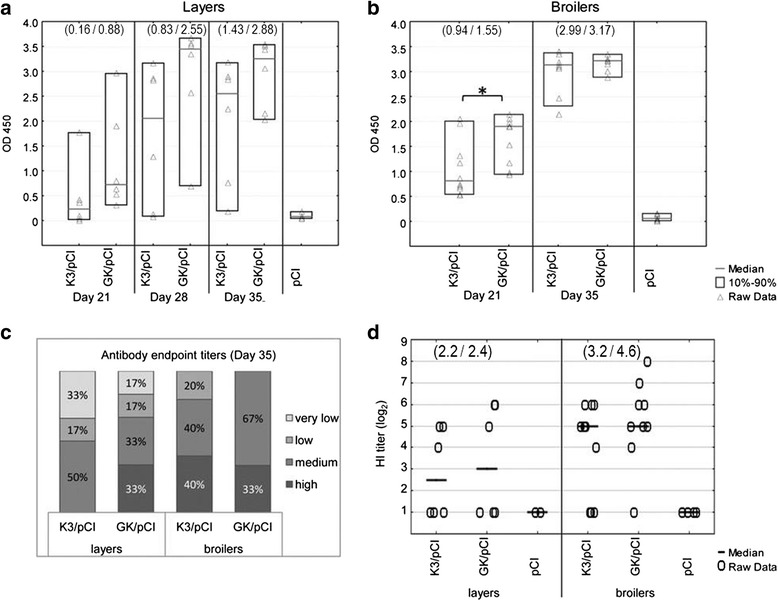
Comparison of humoral responses in sera of chickens immunized with two optimized variants of DNA vaccine. The results for individuals, medians and the 10^th^ and 90^th^ percentiles are shown for K3/pCI and GK/pCI groups of layers (**a**) and broilers (**b**). The percentage distribution of IgY endpoint titers into four categories within the groups and the HI titers are presented in panels (**c**) and (**d**), respectively. The number of individuals was as indicated in Table 1, in Experiment 2 and Experiment 3 for layers and broilers, respectively. The values in the brackets are the geometric means calculated for the K3/pCI and GK/pCI groups

The optimized variants (K3/pCI and GK/pCI) of DNA vaccine were used for immunization of layers and broilers in two independent chicken experiments, labeled as Experiment 2 and 3, respectively. The humoral responses were first evaluated by one-dilution ELISA using the standard 200-fold dilutions of sera (Fig. 6a,b). The medians were always higher in GK/pCI groups than in the respective K3/pCI groups, however only in broilers in sera collected on day 21 (two weeks after the first dose) the differences passed the applied statistical significance test (Fig. 6b).

Next, the endpoint titers of anti-H5 HA in the sera collected two weeks after the booster (on day 35) were assayed and, in order to facilitate the interpretation of data, they were arbitrarily divided into four categories: high (>10^5^), medium (10^4^-10^5^), low (10^3^-10^4^) and very low (<10^3^) (Fig. 6c). In layers, none of the probes from the K3/pCI group reached the end-point titer above 10^5^ and 33 % of probes did not exceed the titer above 10^3^, in contrast to the 33 and 17 % of such probes, respectively, in the case of GK/pCI group. The highest titer of layers’ sera from K3/pCI and GK/pCI group was 7 × 10^4^ and 4 × 10^5^, respectively. The endpoint titers of the broilers’ sera indicated less variability within the groups and the highest titers were more similar (both about 2 × 10^5^). In broilers, 40 % of the probes from the K3/pCI group had titers above 10^5^ and all of them were above 10^3^, while all sera from the GK/pCI group had titers above 10^4^, including 33 % with the titer above 10^5^.

The results of hemagglutination inhibitions (HI) test seem to confirm a slightly better performance of the GK/pCI vaccine over the K3/pCI vaccine in both chicken experiments, however the differences are not statistically significant (Fig. 6d).

## Discussion

DNA vaccines containing the optimized variants of H5 *HA* gene induced strong and specific immune responses in mice and chickens. In both animal models the slight superiority of GK/pCI over K3/pCI was observed. The observed differences were frequently statistically significant. Changes within GK did not regard the codon usage preference in any particular organism but the key was maximization of the GC content at the third coding position of HA (43, 65 and 99.8 % in HAw/pCI, K3/pCI and GK/pCI, respectively). This study was inspired by the previous reports indicating that an increased GC content provides better mRNA stability, processing and nucleocytoplasmic transport [18, 20]. In fact, our results can be explained and are in full agreement with the above literature data. We started with verification of the modified cassettes by monitoring of the level of HA protein produced in mouse muscle cells (C2C12) transfected with K3/pCI and GK/pCI. Indeed, about 15–30 % higher level of HA protein production was observed in cells transfected with GK/pCI than with K3/pCI, which corresponds well with apparently higher immune responses to GK/pCI than to K3/pCI in the immunized animals. The correlation between in vitro expression in transfected cells and immunogenicity of DNA vaccine was also observed by others [24, 25].

Little is known about the timing of cytokine production after immunization. We investigated the profiles of cytokines in the supernatants from the cultured, stimulated with H5 HA for 72h, mice splenocytes that were isolated from the spleens of the immunized animals. In the supernatants we confirmed the presence of five of seven tested cytokines. The levels of IFN-γ, TNF and IL-6 were higher in the group immunized with GK/pCI than with K3/pCI. This result suggests that codon optimization affects both branches of immune responses, humoral (IL-6) and cellular (IFN-γ, TNF) what was previously reported in studies with HIV and HPV DNA vaccine [26, 27]. The lower levels of IL-2 and IL-10 in GK/pCI than K3/pCI group is unclear and need further investigation. The lower level of IL-2 was also observed by Tenbusch et al. in stimulated CD4+ from mice immunized with DNA vaccine containing the optimized sequence for the HA from H1N1 [9]. We did not detect IL-4 nor IL-17A. The lack of IL-17A might be explained by the high concentration of IFN-γ negatively regulating the induction of Th17 cells [28]. The lack of IL-4 might be explained by the conditions of the assay and splenocytes cultivation (and induction) which were optimal for IFN-γ but not IL-4 detection due to the short half-life of the letter [29]. Additional analysis of mice sera (data not shown) indicated that although the level of IgG2a (one of two major isotypes of antibodies) was rather similar in both groups, the level of IgG1 was slightly higher in GK/pCI than in K3/pCI. This result might suggest that the elevation of immune response in GK/pCI group concerned mostly the elevation of Th2 type, however these aspects of the response to the vaccine variants require more studies.

Better efficacy of GK/pCI than K3/pCI observed in chickens is in contrast with the results by Rao et al. [30] who reported that in chicken genome the GC content at the third coding position is negatively correlated with the expression level and that it is not correlated with the maximum expression level. Based on their own analysis, the authors stated that the GC content in genes (general, not only in the third codon positions) could explain only approximately 10 % of the variation in gene expression. According to Kudla at al. [20] the efficient transcription or mRNA processing is responsible for the high expression of GC-rich gene, while other researchers (for example [31, 32]) assumed that the increased expression of codon–optimized genes was caused by the more efficient translational mechanism. The high effectiveness of the variant with nearly 100 % codons with GC at the third position in both model animals (regardless of the codon usage preferences) suggests that the first hypothesis might be correct.

Interestingly, the immunization was generally more effective in broilers than in layers. The results of ELISA test, as well as of HI test were less variable within the broiler groups (70–88 % positives). Immunization of the layers type chickens gave slightly lower and more variable antibody levels and slightly worse results in the HI test (Fig. 5c). On average, the endpoint titers were lower in layers than in broilers, particularly in K3/pCI groups. Many factors can disturb the effectiveness of immunization and the chickens might respond in a very individual way, as observed by others, too [15]. DNA uptake by the target cells, preceded by penetration of sufficient area of tissue after injection seems to be crucial. It is worth to emphasize that the dose chosen for chickens’ immunization was suboptimal for better visualization of the expected differences in immune response. Differences in the strength and dynamic of broilers’ and layers’ responses can be linked to the different genetic background of two used chicken types, differences in their metabolism, growth and development which are results of intensive genetic selection [33]. Chickens are not so popular animal model as mice in immunological studies, still considerable number of publications about chicken immunization with DNA vaccine, especially with avian influenza antigens are available [7]. Most of such experiments were performed with specific pathogen free White Leghorn chickens. In this study we used birds, which are popularly used in Poland for the commercial purposes: the broiler line Ross 308 and the layer line Rosa 1 (a hybrid of Rhode Island and Sussex) and kept them in standard commercial conditions, which allow us to observe natural reactions to the immunizations. Moreover, to our knowledge this is the first report on comparison of the layers’ and the broilers’ humoral responses to the DNA vaccine. Some papers comparing immunological responses of broiler and layer types are available [34–36], however in these experiments conventional vaccines against *Salmonella sp.* were used, or synthetic peptide antigen, not originated from any poultry disease. Similarly to our results, differences in responses between broilers and layers have been previously reported.

## Conclusion

In summary, our results strongly suggest that the enhancement of GC content in the third positions of the codons is a promising strategy for development of DNA vaccine that could be highly effective in a broad range of target species, such as birds and mammals, including humans.

## Additional files

Additional file 1: The mean GC content of the genes in *Homo sapiens*, *Mus musculus*, *Gallus gallus* and Influenza A virus. (PPT 132 kb) Additional file 2: Alignment of sequences encoding H5 HA included in HAw/pCI, K3/pCI and GK/pCI. (DOCX 58 kb) Additional file 3: Serum humoral response in individual mice and chickens after DNA immunization with and without lipofectin. (PPT 157 kb)
