# Supplementary material for: Codon optimization of antigen coding sequences improves the immune potential of DNA vaccines against avian influenza virus H5N1 in mice and chickens
Source: Virol J. 2016 Aug 26;13(1):143. doi: 10.1186/s12985-016-0599-y (PMC5000471; doi:10.1186/s12985-016-0599-y)
Supplement: Additional file 1: — The mean GC content of the genes in Homo sapiens, Mus musculus, Gallus gallus and Influenza A virus. (PPT 132 kb) [file 12985_2016_599_MOESM1_ESM.ppt]

## Slide 1
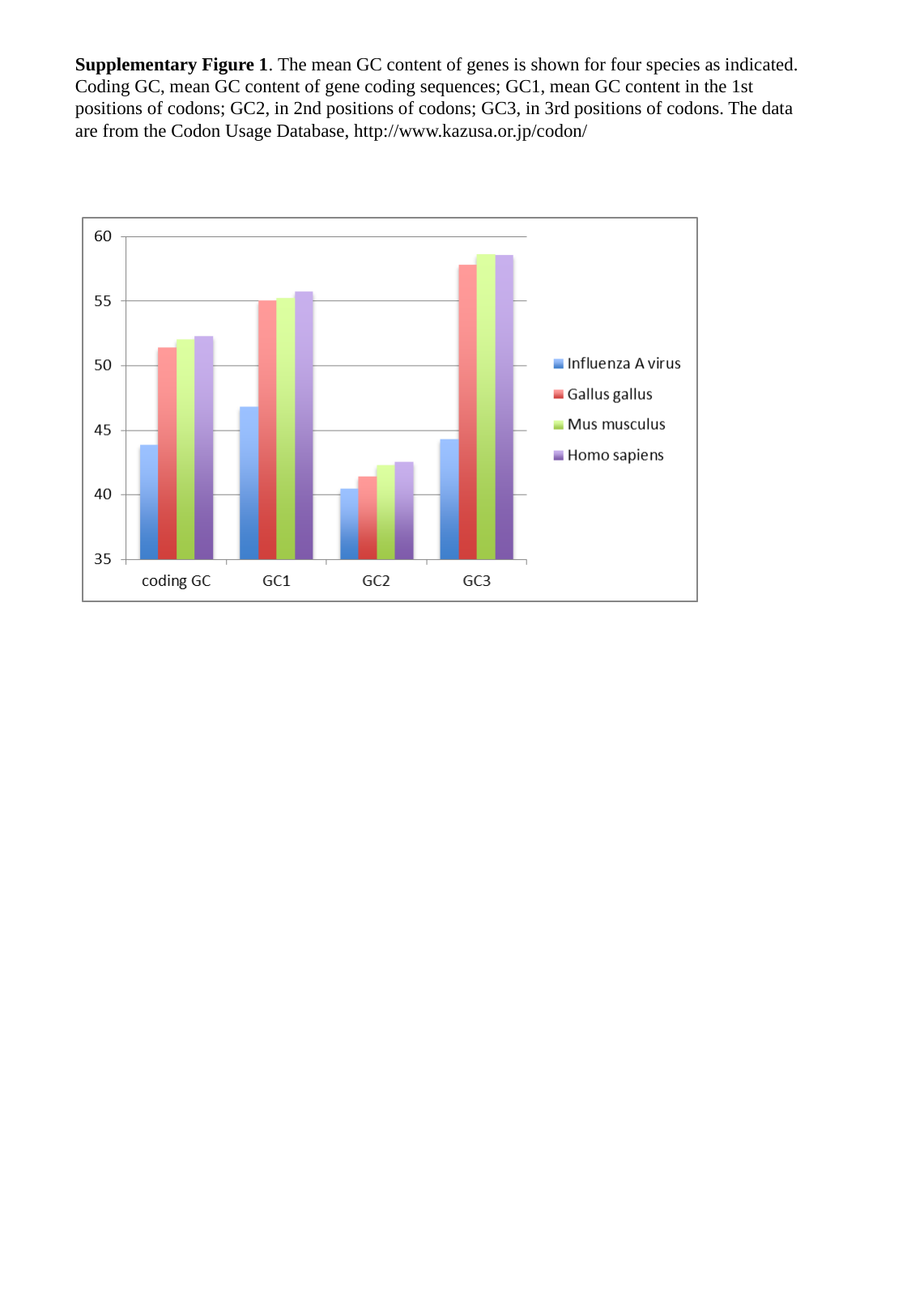

Supplementary Figure 1. The mean GC content of genes is shown for four species as indicated. Coding GC, mean GC content of gene coding sequences; GC1, mean GC content in the 1st positions of codons; GC2, in 2nd positions of codons; GC3, in 3rd positions of codons. The data are from the Codon Usage Database, http://www.kazusa.or.jp/codon/
