# Supplementary material for: Codon optimization of antigen coding sequences improves the immune potential of DNA vaccines against avian influenza virus H5N1 in mice and chickens
Source: Virol J. 2016 Aug 26;13(1):143. doi: 10.1186/s12985-016-0599-y (PMC5000471; doi:10.1186/s12985-016-0599-y)
Supplement: Additional file 2: — Alignment of sequences encoding H5 HA included in HAw/pCI, K3/pCI and GK/pCI. (DOCX 58 kb) [file 12985_2016_599_MOESM2_ESM.docx]

**Supplementary Figure 2**. Alignment of sequences encoding H5 HA included in HAw/pCI (native sequence of A/swan/Poland/305-135V08/2006 (H5H1)), K3/pCI and GK/pCI. All sequences encode the same protein that is indicated above the alignment. The sequence recognized by proteolytic enzymes is in red fonts. The nucleotides different in K3 and GK than in the native sequence (HAw) are highlighted in gray.

**M E K I V L L F A I V S L V K S D Q I C**

HAw ATG GAG AAA ATA GTG CTT CTT TTT GCA ATA GTC AGT CTT GTT AAA AGT GAT CAG ATT TGC

K3 ATG GAA AAG ATT GTG CTG CTG TTT GCT ATC GTG TCC CTG GTG AAA AGC GAC CAG ATT TGT

GK ATG GAG AAG ATC GTG CTG CTG TTC GCC ATC GTG AGC CTG GTC AAG TCC GAC CAG ATC TGC

*** ** ** ** *** ** ** ** ** ** ** ** ** ** ** *** ** **

**I G Y H A N N S T E Q V D T I M E K N V**

HAw ATT GGT TAC CAT GCA AAC AAC TCG ACA GAG CAG GTT GAC ACA ATA ATG GAA AAG AAC GTC

K3 ATC GGG TAT CAT GCC AAT AAC TCA ACC GAA CAG GTG GAT ACT ATT ATG GAG AAA AAC GTG

GK ATC GGC TAC CAC GCC AAC AAC AGC ACC GAG CAG GTG GAC ACC ATC ATG GAG AAG AAC GTG

** ** ** ** ** ** *** ** ** *** ** ** ** ** *** ** ** *** **

**T V T H** **A Q D I L E K T H N G K L C D L**

HAw ACT GTT ACA CAC GCC CAA GAC ATA CTG GAA AAG ACA CAC AAC GGG AAG CTC TGC GAT CTA

K3 ACT GTG ACC CAC GCC CAG GAC ATC CTG GAG AAG ACC CAT AAC GGC AAA CTG TGC GAT CTG

GK ACC GTG ACC CAC GCC CAG GAC ATC CTG GAG AAG ACC CAC AAC GGC AAG CTG TGC GAC CTG

** ** ** *** *** ** *** ** *** ** *** ** ** *** ** ** ** *** ** **

**D G V K P L I L R D C S V A G W L L G N**

HAw GAT GGA GTG AAG CCT CTA ATT TTA AGA GAT TGT AGT GTA GCT GGA TGG CTC CTC GGG AAC

K3 GAC GGA GTG AAG CCC CTG ATC CTG CGC GAT TGC AGC GTG GCT GGC TGG CTG CTG GGA AAC

GK GAC GGC GTG AAG CCC CTG ATC CTG CGC GAC TGC AGC GTG GCC GGC TGG CTG CTG GGC AAC

** ** *** *** ** ** ** * * ** ** ** ** ** ** *** ** ** ** ***

**P M C D E F L N V P E W S Y I V E K I N**

HAw CCA ATG TGT GAC GAA TTC CTC AAT GTG CCG GAA TGG TCT TAC ATA GTG GAG AAG ATC AAT

K3 CCT ATG TGC GAC GAG TTC CTG AAT GTG CCA GAA TGG TCC TAC ATC GTG GAG AAA ATT AAC

GK CCG ATG TGC GAC GAG TTC CTG AAC GTG CCC GAG TGG AGC TAC ATC GTG GAG AAG ATC AAC

** *** ** *** ** *** ** ** *** ** ** *** *** ** *** *** ** ** **

**P A N D** **L C Y P G N F N D Y E E L K H L**

HAw CCA GCC AAT GAC CTC TGT TAC CCA GGG AAT TTC AAC GAC TAT GAA GAA CTG AAA CAC CTA

K3 CCA GCA AAT GAT CTG TGC TAC CCC GGC AAC TTC AAT GAC TAT GAG GAA CTG AAG CAC CTG

GK CCC GCC AAC GAC CTG TGC TAC CCC GGC AAC TTC AAC GAC TAC GAG GAG CTG AAG CAC CTG

** ** ** ** ** ** *** ** ** ** *** ** *** ** ** ** *** ** *** **

**L S R I N H F E K I Q I I P K S S W S D**

HAw TTG AGC AGA ATA AAC CAT TTT GAG AAA ATT CAG ATC ATC CCC AAA AGT TCT TGG TCA GAT

K3 CTG TCT AGG ATC AAC CAT TTC GAA AAG ATC CAG ATC ATC CCT AAG AGC TCC TGG AGC GAT

GK CTG AGC CGG ATC AAC CAC TTC GAG AAG ATC CAG ATC ATC CCC AAG AGC AGC TGG TCC GAC

** * ** *** ** ** ** ** ** *** *** *** ** ** ** *** * *

**H E A S** **S G V S S A C P Y Q G R S S F F**

HAw CAT GAA GCC TCA TCA GGG GTG AGC TCA GCA TGT CCA TAC CAG GGA AGG TCC TCC TTT TTT

K3 CAC GAG GCT TCT TCA GGC GTG AGT AGC GCA TGT CCA TAC CAG GGA CGC TCC TCT TTC TTT

GK CAC GAG GCC AGC AGC GGC GTG AGC AGC GCC TGC CCC TAC CAG GGC CGC AGC TCC TTC TTC

** ** ** ** *** ** ** ** ** *** *** ** * * ** ** **

**R N V V W L I K K D N A Y P T I K R S Y**

HAw AGA AAT GTG GTA TGG CTT ATC AAA AAG GAC AAT GCA TAC CCA ACA ATA AAG AGA AGC TAC

K3 CGG AAC GTG GTG TGG CTG ATT AAG AAA GAC AAT GCT TAC CCA ACT ATC AAA CGC AGC TAT

GK CGG AAC GTG GTG TGG CTG ATC AAG AAG GAC AAC GCC TAC CCC ACC ATC AAG CGC AGC TAC

* ** *** ** *** ** ** ** ** *** ** ** *** ** ** ** ** * *** **

**N N T N Q E D L L V L W G I H H P N D A**

HAw AAT AAT ACC AAC CAA GAA GAT CTT TTG GTA CTG TGG GGG ATT CAC CAT CCA AAT GAT GCG

K3 AAC AAT ACC AAC CAG GAA GAT CTG CTG GTG CTG TGG GGA ATC CAC CAT CCC AAC GAC GCC

GK AAC AAC ACC AAC CAG GAG GAC CTG CTG GTG CTG TGG GGC ATC CAC CAC CCC AAC GAC GCC

** ** *** *** ** ** ** ** ** ** *** *** ** ** *** ** ** ** ** **

**A E Q T R L Y Q N P T T Y I S V G T S T**

HAw GCA GAG CAG ACA AGG CTC TAT CAA AAC CCA ACC ACC TAT ATT TCC GTT GGG ACA TCA ACA

K3 GCT GAG CAG ACA CGG CTG TAC CAG AAT CCT ACC ACA TAT ATT AGT GTG GGG ACA AGC ACT

GK GCC GAG CAG ACC CGC CTG TAC CAG AAC CCC ACC ACC TAC ATC AGC GTG GGC ACC AGC ACC

** *** *** ** * ** ** ** ** ** *** ** ** ** ** ** ** **

**L N Q R** **L V P K I A T R S K V N G Q S G**

HAw CTA AAC CAG AGA TTG GTA CCA AAA ATA GCT ACT AGA TCC AAG GTA AAC GGG CAA AGT GGA

K3 CTG AAC CAG AGA CTG GTG CCC AAG ATC GCA ACA AGG AGC AAA GTG AAT GGC CAG TCC GGA

GK CTG AAC CAG CGG CTG GTG CCC AAG ATC GCC ACC CGC AGC AAG GTG AAC GGC CAG TCC GGC

** *** *** * ** ** ** ** ** ** ** * * ** ** ** ** ** **

**R M E F F W T I L K P N D A I N F E S N**

HAw AGG ATG GAG TTC TTT TGG ACA ATT TTA AAA CCG AAT GAT GCA ATA AAC TTT GAG AGT AAT

K3 AGA ATG GAG TTC TTT TGG ACT ATC CTG AAG CCA AAC GAT GCC ATT AAT TTC GAA TCT AAC

GK CGC ATG GAG TTC TTC TGG ACC ATC CTG AAG CCC AAC GAC GCC ATC AAC TTC GAG TCC AAC

* *** *** *** ** *** ** ** * ** ** ** ** ** ** ** ** ** **

**G N F I A P E N A Y K I V K K G D S T I**

HAw GGA AAT TTC ATT GCT CCA GAA AAT GCA TAC AAA ATT GTC AAG AAA GGG GAC TCA ACA ATT

K3 GGC AAC TTC ATC GCA CCC GAG AAC GCC TAC AAG ATT GTG AAG AAA GGA GAC TCC ACT ATC

GK GGC AAC TTC ATC GCC CCC GAG AAC GCC TAC AAG ATC GTG AAG AAG GGC GAC AGC ACC ATC

** ** *** ** ** ** ** ** ** *** ** ** ** *** ** ** *** ** **

**M K S E L E Y G N C N T K C Q T P I G A**

HAw ATG AAA AGT GAA TTG GAA TAT GGT AAC TGC AAC ACC AAG TGT CAA ACT CCA ATA GGG GCG

K3 ATG AAA TCT GAG CTG GAA TAT GGG AAC TGC AAT ACC AAG TGT CAG ACA CCT ATC GGT GCA

GK ATG AAG AGC GAG CTG GAG TAC GGC AAC TGC AAC ACC AAG TGC CAG ACC CCC ATC GGC GCC

*** ** ** ** ** ** ** *** *** ** *** *** ** ** ** ** ** ** **

**I N S S** **M P F H N I H P L T I G E C P K**

HAw ATA AAC TCT AGT ATG CCA TTC CAC AAC ATC CAC CCT CTC ACC ATC GGG GAA TGC CCC AAA

K3 ATT AAC TCA AGT ATG CCC TTT CAC AAT ATC CAT CCT CTG ACC ATT GGG GAG TGC CCC AAG

GK ATC AAC TCC AGC ATG CCC TTC CAC AAC ATC CAC CCC CTC ACC ATC GGC GAG TGC CCC AAG

** *** ** ** *** ** ** *** ** *** ** ** ** *** ** ** ** *** *** **

**Y V K S** **N R L V L A T G L R N S P Q G E**

HAw TAT GTG AAA TCA AAC AGA TTA GTC CTT GCG ACT GGG CTC AGA AAT AGC CCT CAA GGA GAG

K3 TAC GTG AAA AGT AAC CGC CTG GTG CTG GCC ACA GGT CTG CGG AAT AGC CCT CAG GGG GAA

GK TAC GTG AAG AGC AAC CGC CTG GTG CTG GCC ACC GGC CTG CGG AAC TCC CCC CAG GGC GAG

** *** ** *** * * ** ** ** ** ** ** * ** * ** ** ** **

**R R R K K R G L F G A I A G F I E G G W**

HAw AGA AGA AGA AAA AAG AGA GGA CTA TTT GGA GCT ATA GCA GGT TTT ATA GAG GGA GGA TGG

K3 --- --- --- --- --- --- GGT CTG TTC GGG GCT ATC GCA GGT TTT ATT GAG GGC GGA TGG

GK --- --- --- --- --- --- GGC CTG TTC GGC GCC ATC GCC GGC TTC ATC GAG GGC GGC TGG

** ** ** ** ** ** ** ** ** ** *** ** ** ***

**Q G M V D G W Y G Y H H S N E Q G S G Y**

HAw CAG GGA ATG GTA GAT GGT TGG TAT GGG TAC CAC CAT AGC AAC GAG CAG GGG AGT GGG TAC

K3 CAG GGA ATG GTG GAT GGG TGG TAC GGT TAT CAC CAT TCA AAC GAA CAG GGC AGT GGA TAC

GK CAG GGC ATG GTG GAC GGC TGG TAC GGC TAC CAC CAC AGC AAC GAG CAG GGC AGC GGC TAC

*** ** *** ** ** ** *** ** ** ** *** ** *** ** *** ** ** ** ***

**A A D K E S T Q K A I D G V T N K V N S**

HAw GCT GCA GAC AAA GAA TCC ACT CAA AAG GCA ATA GAT GGA GTC ACC AAT AAG GTC AAC TCG

K3 GCA GCC GAT AAG GAG TCA ACA CAG AAA GCC ATT GAC GGA GTG ACT AAC AAG GTG AAC TCC

GK GCC GCC GAC AAG GAG AGC ACC CAG AAG GCC ATC GAC GGC GTG ACC AAC AAG GTG AAC AGC

** ** ** ** ** ** ** ** ** ** ** ** ** ** ** *** ** ***

**I I N K** **M N T Q F E A V G R E F N N L E**

HAw ATC ATT AAC AAA ATG AAC ACT CAG TTT GAG GCC GTT GGA AGG GAA TTT AAT AAC TTA GAA

K3 ATC ATT AAC AAA ATG AAC ACC CAG TTC GAG GCT GTG GGG AGA GAG TTC AAC AAT CTG GAG

GK ATC ATC AAC AAG ATG AAC ACC CAG TTC GAG GCC GTG GGC CGC GAG TTC AAC AAC CTG GAG

*** ** *** ** *** *** ** *** ** *** ** ** ** * ** ** ** ** * **

**P R I E N L N K K M E D G F L D V W T Y**

HAw AGG AGA ATA GAA AAT TTA AAC AAG AAG ATG GAA GAC GGA TTC CTA GAT GTC TGG ACT TAT

K3 AGA AGG ATC GAA AAC CTG AAT AAG AAA ATG GAA GAT GGC TTC CTG GAC GTG TGG ACT TAC

GK CGG CGC ATC GAG AAC CTG AAC AAG AAG ATG GAG GAC GGC TTC CTG GAC GTG TGG ACC TAC

* * ** ** ** * ** *** ** *** ** ** ** *** ** ** ** *** ** **

**N A E L L V L M E N E R T L D F H D S N**

HAw AAT GCT GAA CTT CTG GTT CTC ATG GAA AAT GAG AGA ACT CTA GAC TTT CAT GAC TCA AAT

K3 AAC GCT GAG CTG CTG GTG CTG ATG GAG AAT GAA AGG ACC CTG GAT TTT CAC GAC AGC AAC

GK AAC GCC GAG CTG CTG GTG CTC ATG GAG AAC GAG CGC ACC CTG GAC TTC CAC GAC AGC AAC

** ** ** ** *** ** ** *** ** ** ** * ** ** ** ** ** *** **

**V K N L Y D K V R L Q L R D N A K E L G**

HAw GTC AAG AAC CTT TAC GAC AAG GTC CGA CTA CAG CTT AGG GAT AAT GCA AAG GAG CTT GGT

K3 GTG AAG AAT CTG TAT GAT AAA GTG AGA CTG CAG CTG AGG GAC AAC GCA AAG GAA CTG GGG

GK GTG AAG AAC CTG TAC GAC AAG GTG CGC CTG CAG CTG CGG GAC AAC GCC AAG GAG CTC GGT

** *** ** ** ** ** ** ** * ** *** ** ** ** ** ** *** ** ** **

**N G C F E F Y H R C D N E C M E S V R N**

HAw AAC GGT TGT TTC GAG TTC TAT CAC AGA TGT GAT AAT GAA TGC ATG GAA AGT GTA AGA AAC

K3 AAT GGT TGT TTC GAG TTT TAC CAT AGA TGC GAT AAC GAG TGT ATG GAA TCC GTG AGG AAT

GK AAC GGC TGC TTC GAG TTC TAC CAC CGG TGC GAC AAC GAG TGC ATG GAG AGC GTG CGC AAC

** ** ** *** *** ** ** ** * ** ** ** ** ** *** ** ** * **

**G T Y D Y P Q Y S E E A R L K R E E I S**

HAw GGA ACG TAT GAC TAC CCG CAG TAT TCA GAA GAA GCA AGA TTA AAA AGA GAG GAA ATA AGT

K3 GGC ACA TAC GAC TAT CCA CAG TAT TCT GAG GAA GCC CGC CTG AAG CGG GAG GAA ATT TCT

GK GGC ACC TAC GAC TAC CCC CAG TAC TCC GAG GAG GCC CGC CTG AAG CGG GAG GAG ATC AGC

** ** ** *** ** ** *** ** ** ** ** ** * * ** * *** ** **

**G V K L E S I G T Y Q I L S I Y S T V A**

HAw GGA GTA AAA TTG GAA TCA ATA GGA ACC TAC CAA ATA CTG TCA ATT TAT TCA ACA GTG GCG

K3 GGG GTG AAA CTG GAG TCA ATC GGT ACC TAC CAG ATC CTG TCT ATC TAC TCA ACA GTG GCT

GK GGC GTG AAG CTG GAG AGC ATC GGC ACC TAC CAG ATC CTG AGC ATC TAC TCC ACC GTG GCG

** ** ** ** ** ** ** *** *** ** ** *** ** ** ** ** *** **

**S S L A L A I M V A G L S L W M C S N G**

HAw AGC TCC CTA GCA CTG GCA ATC ATG GTG GCT GGT CTA TCT TTA TGG ATG TGC TCC AAT GGA

K3 AGC TCC CTG GCC CTG GCT ATC ATG GTG GCT GGC CTG AGC CTG TGG ATG TGC TCT AAC GGT

GK AGC AGC CTG GCC CTG GCC ATC ATG GTG GCC GGC CTG TCC CTG TGG ATG TGC AGC AAC GGC

*** * ** ** *** ** *** *** *** ** ** ** * *** *** *** ** **

**S L Q C R I C I ***

HAw TCG TTA CAA TGC AGA ATT TGC ATT TAA

K3 AGC CTG CAG TGT AGG ATC TGT ATT TGA

GK TCC CTG CAG TGC CGC ATC TGC ATC TAA

* ** ** * ** ** ** * *
