# Supplementary material for: Codon optimization of antigen coding sequences improves the immune potential of DNA vaccines against avian influenza virus H5N1 in mice and chickens
Source: Virol J. 2016 Aug 26;13(1):143. doi: 10.1186/s12985-016-0599-y (PMC5000471; doi:10.1186/s12985-016-0599-y)
Supplement: Additional file 3: — Serum humoral response in individual mice and chickens after DNA immunization with and without lipofectin. (PPT 157 kb) [file 12985_2016_599_MOESM3_ESM.ppt]

## Slide 1
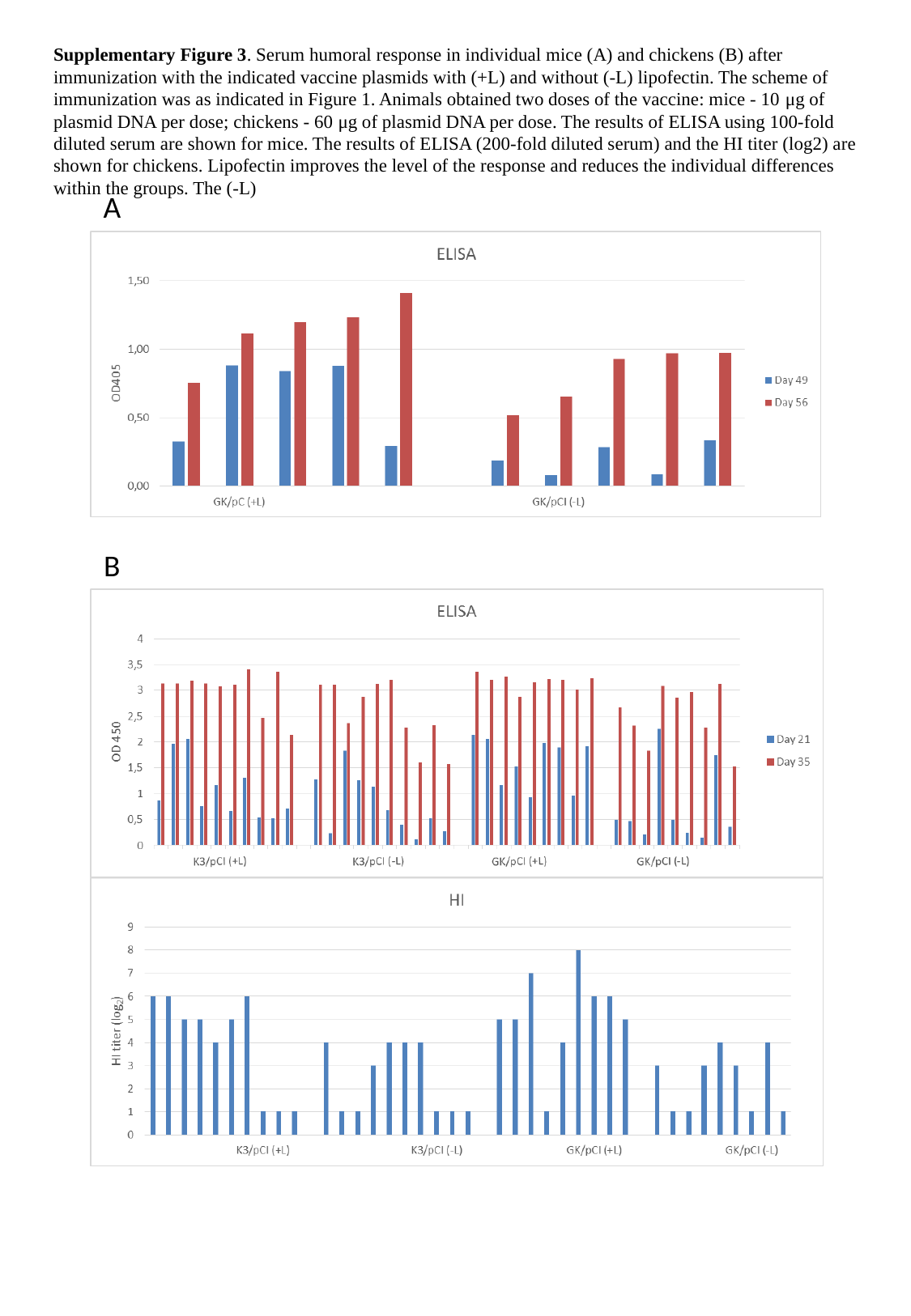

Supplementary Figure 3. Serum humoral response in individual mice (A) and chickens (B) after immunization with the indicated vaccine plasmids with (+L) and without (-L) lipofectin. The scheme of immunization was as indicated in Figure 1. Animals obtained two doses of the vaccine: mice - 10 μg of plasmid DNA per dose; chickens - 60 μg of plasmid DNA per dose. The results of ELISA using 100-fold diluted serum are shown for mice. The results of ELISA (200-fold diluted serum) and the HI titer (log2) are shown for chickens. Lipofectin improves the level of the response and reduces the individual differences within the groups. The (-L)
A
B
